# Genome sequence and characterization of *Streptomyces* phages Vanseggelen and Verabelle, representing two new species within the genus *Camvirus*

**DOI:** 10.1038/s41598-023-47634-3

**Published:** 2023-11-17

**Authors:** Véronique Ongenae, Annabel Kempff, Vera van Neer, Helena Shomar, Florian Tesson, Daniël Rozen, Ariane Briegel, Dennis Claessen

**Affiliations:** 1 Molecular Biotechnology, Institute of Biology, Leiden University, P.O. Box 9505, 2300 RA Leiden, The Netherlands; 2 Centre for Microbial Cell Biology, Leiden University, Leiden, The Netherlands; 3 MDM Lab, Department Genomes and Genetics, Pasteur Institute, Paris, France; 4 INSERM, U1284, Université Paris-Cité, Paris, France

**Keywords:** Microbiology, Bacteriophages

## Abstract

Despite the rising interest in bacteriophages, little is known about their infection cycle and lifestyle in a multicellular host. Even in the model system *Streptomyces*, only a small number of phages have been sequenced and well characterized so far. Here, we report the complete characterization and genome sequences of *Streptomyces* phages Vanseggelen and Verabelle isolated using *Streptomyces coelicolor* as a host. A wide range of *Streptomyces* strains could be infected by both phages, but neither of the two phages was able to infect members of the closely related sister genus *Kitasatospora*. The phages Vanseggelen and Verabelle have a double-stranded DNA genome with lengths of 48,720 and 48,126 bp, respectively. Both phage genomes contain 72 putative genes, and the presence of an integrase encoding protein indicates a lysogenic lifestyle. Characterization of the phages revealed their stability over a wide range of temperatures (30–45 °C) and pH values (4–10). In conclusion, *Streptomyces* phage Vanseggelen and *Streptomyces* phage Verabelle are newly isolated phages that can be classified as new species in the genus *Camvirus,* within the subfamily *Arquattrovirinae.*

## Introduction

*Streptomyces* are Gram-positive, spore-forming bacteria, with a relatively high G + C-content^1^. Their multicellular morphology and life cycle are unique within the bacteria realm. Furthermore, *Streptomyces* are well-known for their ability to produce natural products with a wide range of biological activities^2^. These include, for example, metabolites that can act as anti-fungals, anti-virals, anti-tumor agents and many clinically relevant antibiotics^3^. However, the production of these metabolites from *Streptomyces* can be negatively influenced by bacteriophage contaminations, which can result in huge economic losses^4^. Bacteriophages, or phages for short, are viruses that can infect bacteria. Yet, it remains largely unknown how bacteriophages can recognize, attach and infect these multicellular bacteria and more detailed descriptions of new phage species will be beneficial for following reseach^5,6^. All known *Streptomyces* phages belong to the class *Caudoviricetes,* which are tailed bacterial and archaeal viruses with icosahedral capsid heads and double-stranded DNA genomes^7^. The phages within this class are divided into three morphotypes: long contractile tails (myoviruses), long non-contractile tails (siphoviruses) or short non-contractile tails (podoviruses). Of all characterized *Streptomyces* phages, 91% have long non-contractile tails and are therefore siphoviruses^8^. Compared to other genera, relatively few *Streptomyces* phages have been properly characterized and sequenced. According to The Actinobacteriophage Database^9^, only 340 *Streptomyces* phages have been sequenced to date, while there are over 2700 *Escherichia coli* phage genomes deposited in Genbank^10,11^.

Here, we describe two newly discovered Actinobacteriophages, which we name *Streptomyces* phage Vanseggelen and *Streptomyces* phage Verabelle. These temperate phages belong to the genus *Camvirus*, within the subfamily *Arquattrovirinae*. Vanseggelen has an icosahedral capsid head of 53.7 nm ± 7.9 nm and a long non-contractile tail of 228.7 ± 7.5 nm, while Verabelle has an icosahedral capsid head of 63.2 + 1.2 nm and a long non-contractile tail of 200.4 + 1.5 nm. Furthermore, we provide a complete genomic analysis of these newly identified phages. Together, these results will hopefully increase our knowledge on phage-host dynamics in *Streptomyces* in the future and expanded the list of available, sequenced and characterized *Streptomyces* phages.

## Materials and methods

### Bacteriophage isolation

*Streptomyces* phages Vanseggelen and Verabelle were isolated from a soil sample obtained from the National Park Zuid-Kennemerland in the Netherlands (N52° 23′ 31″, E4° 34′ 49″) using *Streptomyces coelicolor* as the host. Details on isolation procedures are reported previously in detail^12^. The collected phage stock solutions were stored at 4 °C.

### Host range analysis

The host range of Vanseggelen and Verabelle was determined by using serial dilutions on double agar overlay plates. The phage stock solutions were serial diluted with Difco Nutrient Broth (DNB) (BD biosciences) supplemented with 4 mM Ca(NO_3_)_2_ and 0.5% w/v glucose. Droplets of 3 µl of the phage dilutions were spotted in duplicate on a bacterial lawn of nine different *Streptomyces strains* and one *Kitasatospora* strain from the Microbial Biotechnology (MBT) collection at Leiden University^13^. *Streptomyces* strains MBT13, MBT61 and MBT86 are new species and have not been taxonomically classified yet. The plates were incubated at 30 °C for at least 24 h before lysis was observed, after which the presence of a lysis zone was scored as a positive result. The efficiency of plating (EOP) for a given strain was calculated relative to the host strain *S. coelicolor*, only when distinct clear individual plaques were observed.

### Morphology analysis

The plaque morphology of Vanseggelen and Verabelle was determined by using serial dilutions on double agar overlay plates on the host *S. coelicolor.*

Representative images of the phages were made using transmission electron microscopy (TEM). For a single grid preparation, 3 µl of the phage lysates (Vanseggelen = 10^6^ PFU/ml and Verabelle = 10^7^ PFU/ml) was placed on a glow-discharged 200 mesh carbon coated copper grid (EMS) and allowed to set for 30 s before excess sample solution was removed by filter paper. The phages were stained with 2% uranyl acetate for 45 s after which excess liquid was removed and the samples were air-dried for an additional 30 min. The grids were observed using a single tilt specimen holder inside a 120 kV Talos L120C TEM with a Lab6 electron source and Ceta detector at the Netherlands Center for Nanoscopy (NeCEN, Leiden).

### Stability analysis

A one-step growth curve analysis was performed in triplicate to determine the latent period and burst size of both phages^14^. Since *Streptomyces* phage adsorption was found to be maximal for germlings^15^, 10^8^ spores ml^−1^ of *S. coelicolor* were allowed to germinate in 10 ml DNB medium at 30 °C while shaking at 200 rpm. After approximately 5 h, the culture was infected with Vanseggelen or Verabelle at Multiplicity of Infection (MOI) of 0.01 and 100 µl was sampled at 10 min intervals up to 180 min. The samples were filtered with a 0.20 µm filter, serial diluted with DNB medium and immediately plated on double agar overlay plates. The burst size was calculated using the following formula:

$$ \begin{aligned} & {\text{Burst}}\;{\text{size}} = {\text{average}}\;{\text{of}}\;{\text{free}}\;{\text{phages}}\;{\text{after}}\;{\text{burst}}\left( {{\text{T}} = {15}0\;{\text{to}}\;{\text{T}} = {18}0} \right) \\ & - {\text{average}}\;{\text{of}}\;{\text{free}}\;{\text{phages}}\;{\text{before}}\;{\text{burst}}\left( {{\text{T}} = {1}0\;{\text{to}}\;{\text{T}} = {14}0} \right)/{\text{number}}\;{\text{of}}\;{\text{phages}}\;{\text{at}}\;{\text{T}} = 0 \\ \end{aligned} $$

The thermal stability of Vanseggelen and Verabelle was determined by adding 100 µl of phage suspension (Vanseggelen = 10^5^ PFU/ml and Verabelle = 10^7^ PFU/ml) to 900 µl DNB medium and incubating the phages at 25, 30, 37, 45, 55 and 65 °C. The samples were filtered with a 0.20 µm filter after 1 h of incubation. A serial dilution was made with DNB medium, which was immediately spotted on double agar overlay plates to determine the phage titers. To assess viability at common storage temperatures, the phages were incubated in DNB medium without glycerol at -80, -20 and 4 °C for seven days. To determine pH stability, 100 µl of the phage suspensions was added to 900 µl DNB medium that was pH adjusted using 1 M HCl or 1 M NaOH. The samples were incubated at 30 °C for 1 h before filtration. A serial dilution was made with DNB medium, which was immediately spotted on double agar overlay plates to determine the phage titer. All the phage stability measurements were performed in triplicate. All statistical analyses were performed with a Student’s *t*-test.

### DNA extraction and phylogenetic analysis

DNA isolation, whole genome sequencing and de novo assembly of Vanseggelen and Verabelle were performed by the Institute Pasteur using Illumina NovaSeq PE150 sequencing (Paris, France). The complete genome sequences of Vanseggelen and Verabelle were deposited at GenBank under accession number OQ970438 and OQ970439, respectively. Linear maps of the genomes were constructed by Geneious Prime 2022.1.1 (https://www.geneious.com) and genomes were de novo assembled using SPAdes v3.15.5 and aligned starting by the phage terminase^16^.

A viral proteomic phylogenetic tree and a genomic alignment based on the whole genome sequences of *Streptomyces* phages Alsaber, Amela, Endor1, Endor2, Hydra, Indigo, Joe, Pablito, phiCAM, Saftant, Sitrop, Verse and Yosif were constructed using ViPtree: the Viral Proteomic Tree version 3.5^17^. The genome sequences of these phages were acquired from the National Center for Biotechnology Information (NCBI). Pairwise genome comparisons of phages within the *Camvirus* genus were visualized and performed with clinker^18^. A heatmap to determine intergenomic relatedness was constructed with the use of VIRIDIC^19^.

## Results

### Morphology and host range

**Figure 1:**
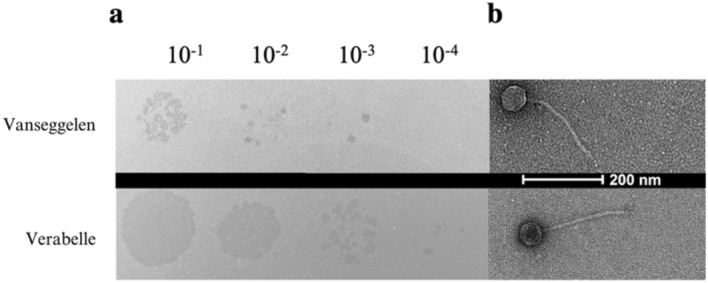
Morphology of Vanseggelen and Verabelle. (**a**) Plaque morphology after 24 h of infection in *S. coelicolor*, using a serial dilution with the DNB double agar overlay method. (**b**) Representative TEM images of Vanseggelen and Verabelle stained with 2% uranyl acetate.

Two novel phages infecting *Streptomyces* have been isolated from soil samples in the Netherlands. Both phages produced small, clear plaques on the host strain *S. coelicolor* while using the double agar overlay method (Fig. 1a). TEM images revealed that Vanseggelen has an icosahedral capsid head of 53.7 nm ± 7.9 nm (n = 5) and a long non-contractile tail of 228.7 ± 7.5 nm (n = 4). Verabelle has an icosahedral capsid head of 63.2 ± 1.2 nm (n = 3) and a long non-contractile tail of 200.4 ± 1.5 nm (n = 3) (Fig. 1b).

A host range analysis of Verabelle performed on ten different actinomycetes strains revealed that all nine *Streptomyces* strains could be infected, and the phage produced clear as well as turbid lysis zone morphologies depending on the host (Table 1). Vanseggelen was not able to infect *Streptomyces griseus*, *Streptomyces lividans* and MBT13, but could infect the other six *Streptomyces* strains tested (Table 2). However, both phages were unable to infect *K. virifidaciens*. When distinct clear individual plaques were visible in a given strain, the efficiency of plating (EOP) was determined relative to the original host *S. coelicolor.*

**Table 1:** Host range determination of *Streptomyces* phage Verabelle.

Bacterial strain	Origin	Infectivity	Lysis zone morphology	EOP
*Streptomyces coelicolor*	MBT collection	+	Clear	1.0
*Streptomyces albus*	MBT collection	+	Clear	40.9
*Streptomyces cattleya*	MBT collection	+	Clear	4.1
*Streptomyces griseus*	MBT collection	+	Turbid	
*Streptomyces lividans*	MBT collection	+	Turbid	
*Streptomyces venezuelae*	MBT collection	+	Turbid	
*Kitasatospora virifidaciens*	DSM40239	−	None	
MBT13	MBT collection	+	Turbid	
MBT61	MBT collection	+	Clear	9.5
MBT86	MBT collection	+	Clear	2.2

The susceptibility of different actinomycetes strains to infection by Verabelle, determined by performing plaque assays with serial dilutions. A clear lysis zone morphology was scored positive when distinct single plaques could be observed.

**Table 2:** Host range determination of *Streptomyces* phage Vanseggelen.

Bacterial strain	Origin	Infectivity	Lysis zone morphology	EOP
*Streptomyces coelicolor*	MBT collection	+	Clear	1.0
*Streptomyces albus*	MBT collection	+	Clear	3.2
*Streptomyces cattleya*	MBT collection	+	Clear	1.7
*Streptomyces griseus*	MBT collection	−	None	
*Streptomyces lividans*	MBT collection	−	None	
*Streptomyces venezuelae*	MBT collection	+	Turbid	
*Kitasatospora virifidaciens*	DSM40239	−	None	
MBT13	MBT collection	−	None	
MBT61	MBT collection	+	Clear	3.2
MBT86	MBT collection	+	Clear	1.5

The susceptibility of different actinomycetes strains to infection by Vanseggelen, determined by performing plaque assays with serial dilutions. A clear lysis zone morphology was scored positive when distinct single plaques could be observed.

### One-step growth curve

**Figure 2:**
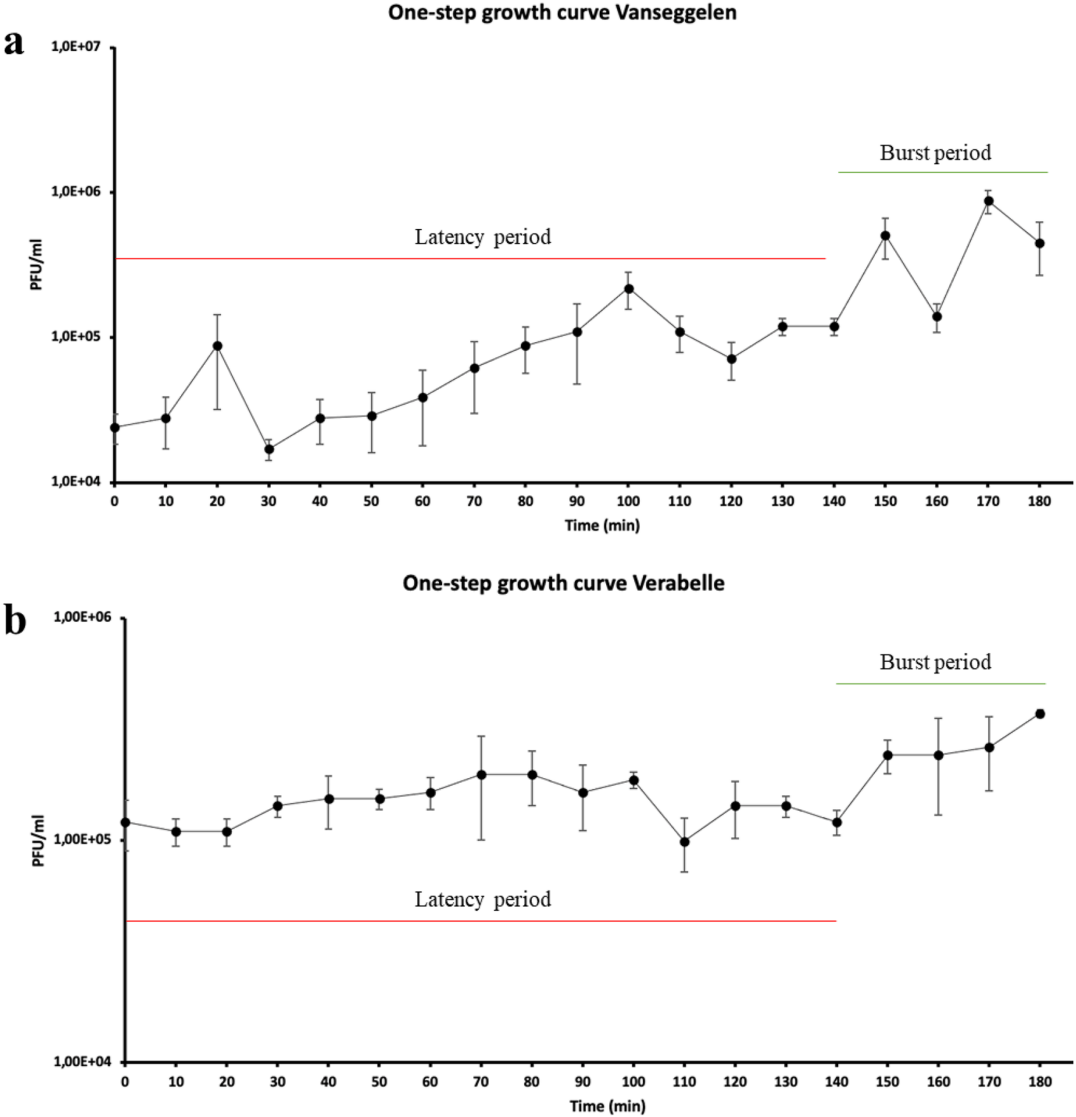
One-step growth curves of Vanseggelen and Verabelle at MOI = 0.01. (**a**) Vanseggelen had a latent period of 140 min and a total burst size of 17 virions per cell. (**b**) Verabelle had a latent period of 140 min and a total burst size of 8 virions per cell.

The one-step growth curve of Vanseggelen shows a latent period of 140 min, with a burst size of 17 virions per bacterial cell (Fig. 2a). The one-step growth curve of Verabelle also shows a latent period of 140 min, but with a burst size of 8 virions per bacterial cell (Fig. 2b). The latent period of both phages is relatively long compared to other *Streptomyces* phages^12,20^.

### Phage stability

**Figure 3:**
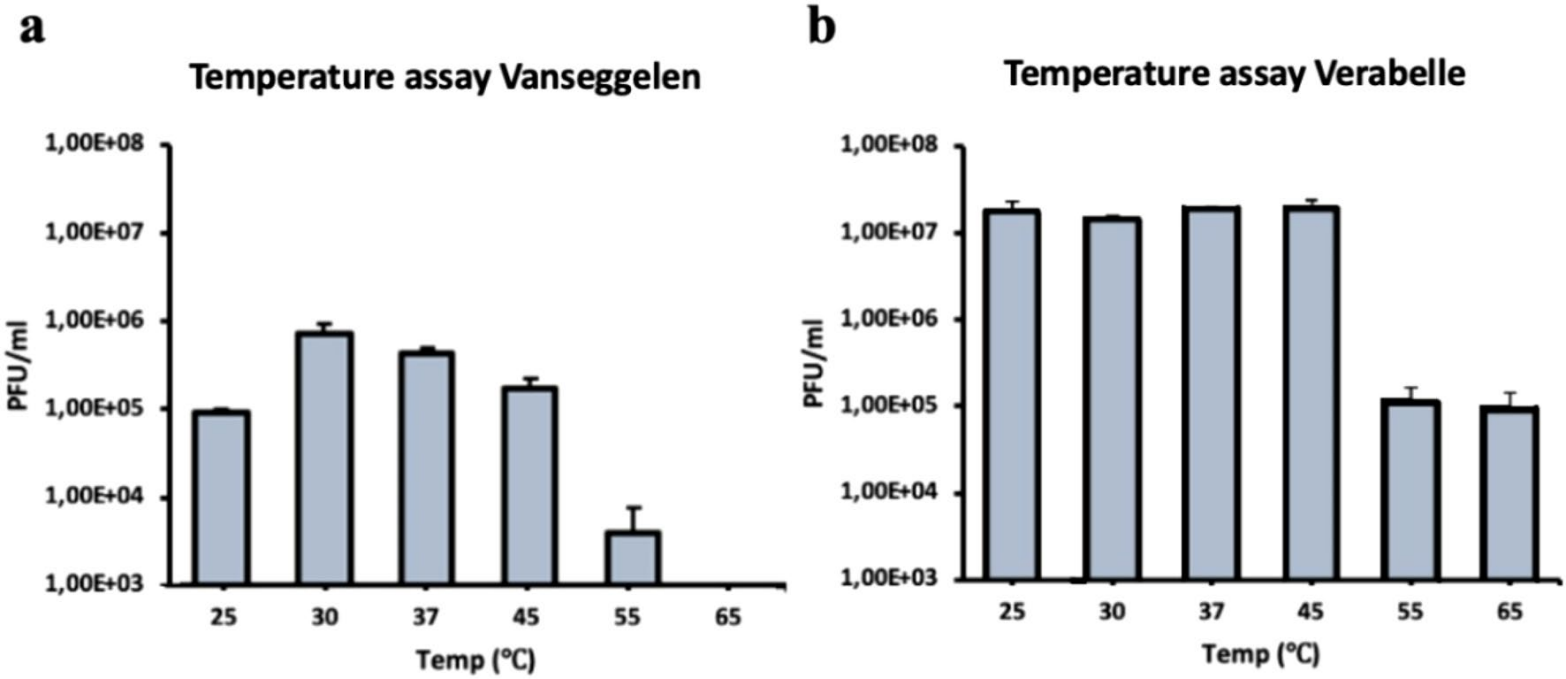
Temperature stability of Vanseggelen and Verabelle. (**a**) Vanseggelen was stable from 30 up to 45 °C without a reduction in viability. However, the infectivity significantly decreased when the phage was incubated for one hour at 55 °C or higher. (**b**) Verabelle remains stable between temperatures ranging from 25 to 45 °C, but the viability significantly decreased at higher temperatures.

**Figure 4:**
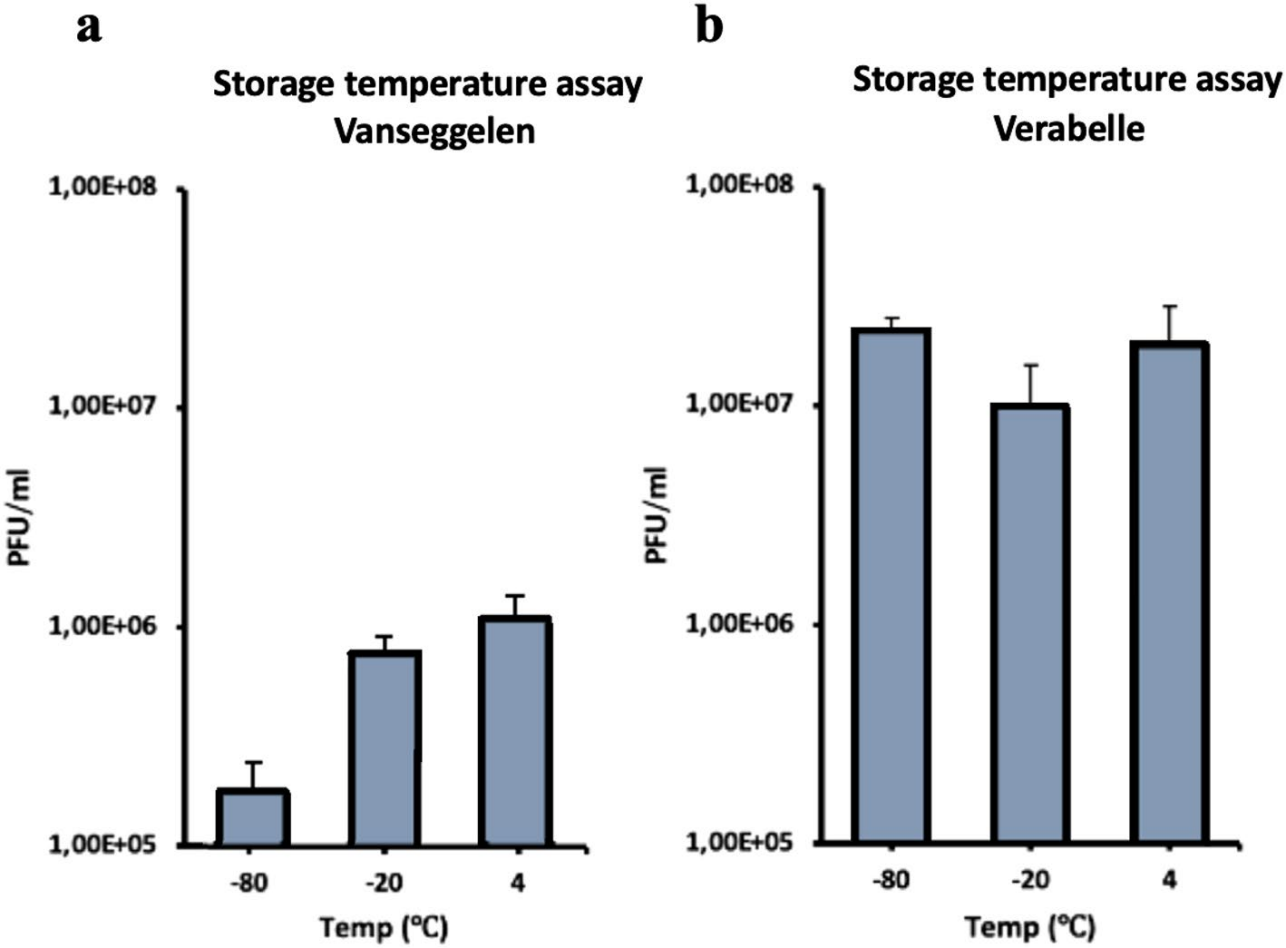
Storage stability of Vanseggelen and Verabelle (**a**) Vanseggelen retains its viability when stored at − 20 or 4 °C (**b**) Verabelle remains viable at all storage temperatures.

**Figure 5:**
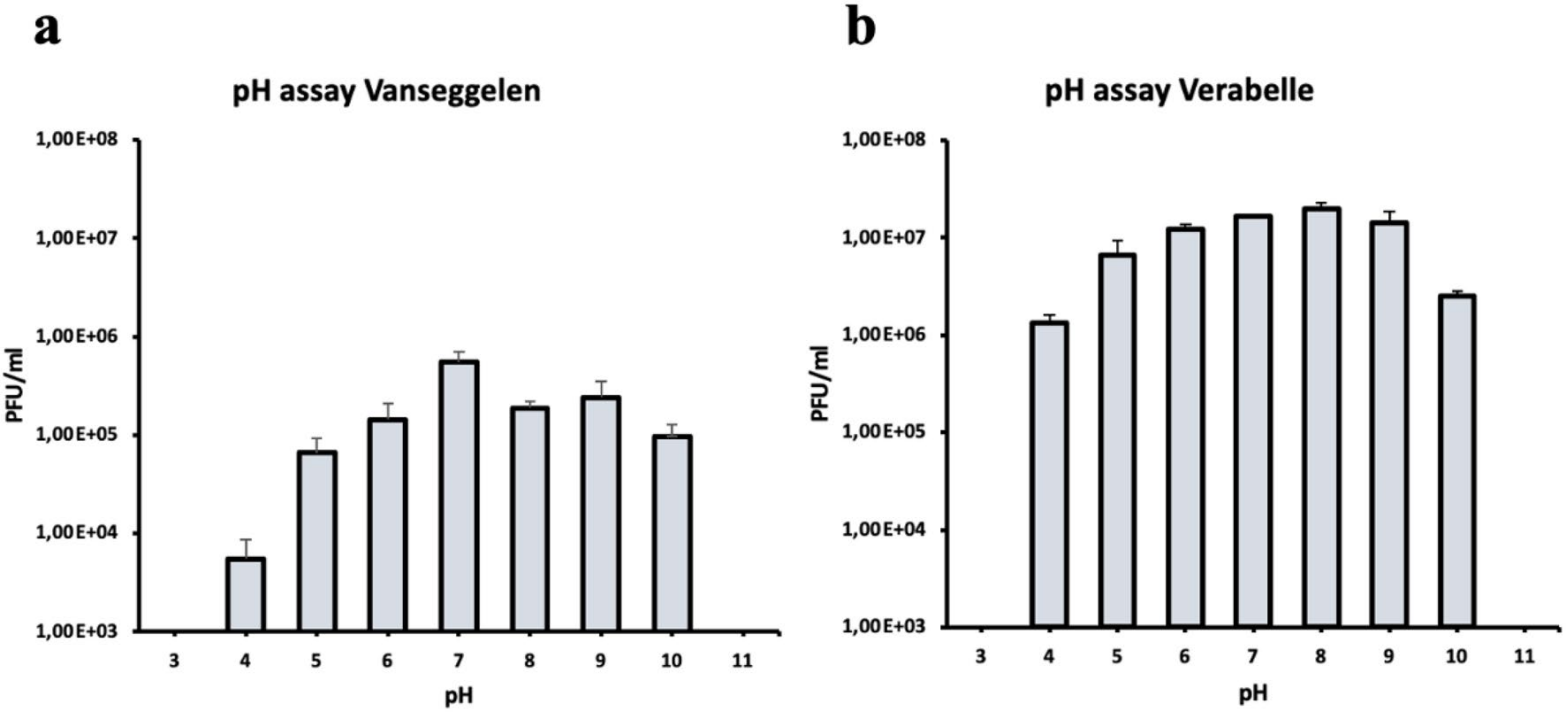
pH stability of Vanseggelen and Verabelle. (**a**) Vanseggelen remains relatively stable at pH values 5.0–10.0. (**b**) Verabelle remains relatively stable at pH values 4.0–10.0 and shows no viability at pH values 3.0 and 11.0.

Vanseggelen and Verabelle were stable up to 45 °C without a significant reduction in viability (Fig. 3). The absence of plaques at 65 °C indicates that Vanseggelen was not viable after incubation at 65 °C for 1 h. In addition, Vanseggelen made significantly less plaques after storing the phage samples at − 80 °C for one week (Fig. 4a), while Verabelle could be incubated at temperatures of − 80, − 20 and 4 °C without loss in viability (Fig. 4b). The optimal pH range for Vanseggelen was between pH 6.0–10.0, from which a decreasing trend in viability was visible at both lower and higher acidity (Fig. 5a). Verabelle remained relatively stable at pH values from 4.0 to 10.0 and no viability was detected at pH values of 3.0 and 11.0 (Fig. 5b).

### Genomic analysis

**Figure 6:**
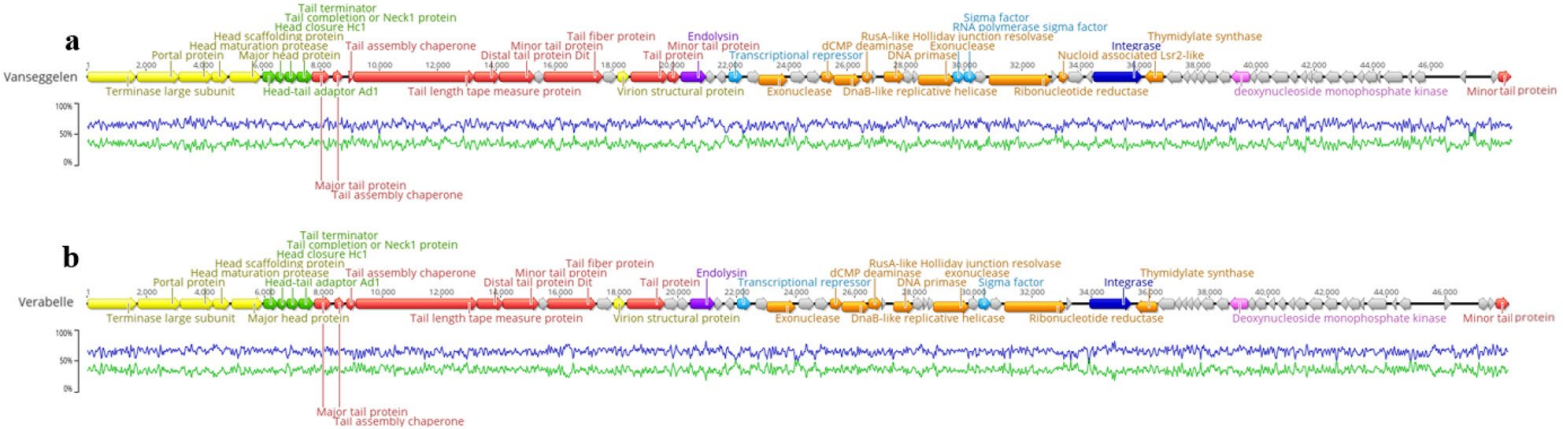
Schematic representation of the double-stranded DNA sequence of Vanseggelen and Verabelle. (**a**) Overview of the linear genome of Vanseggelen. (**b**) Overview of the linear genome of Verabelle. (**a**)**/**(**b**) Arrowheads are indicated in the direction of the coding sequence (CDS). Hypothetical proteins are displayed in grey and the predicted proteins are color coded as follows: yellow for head and packaging, red for tail, orange for DNA, RNA and nucleotide metabolism, green for connector, purple for lysis, light blue for transcription regulation, dark blue for integration and excision and pink for other. The blue line represents the GC content, while the green line represents the AT content. The genome maps were generated through Geneious Prime 2022.1.1. (https://www.geneious.com).

**Figure 7:**
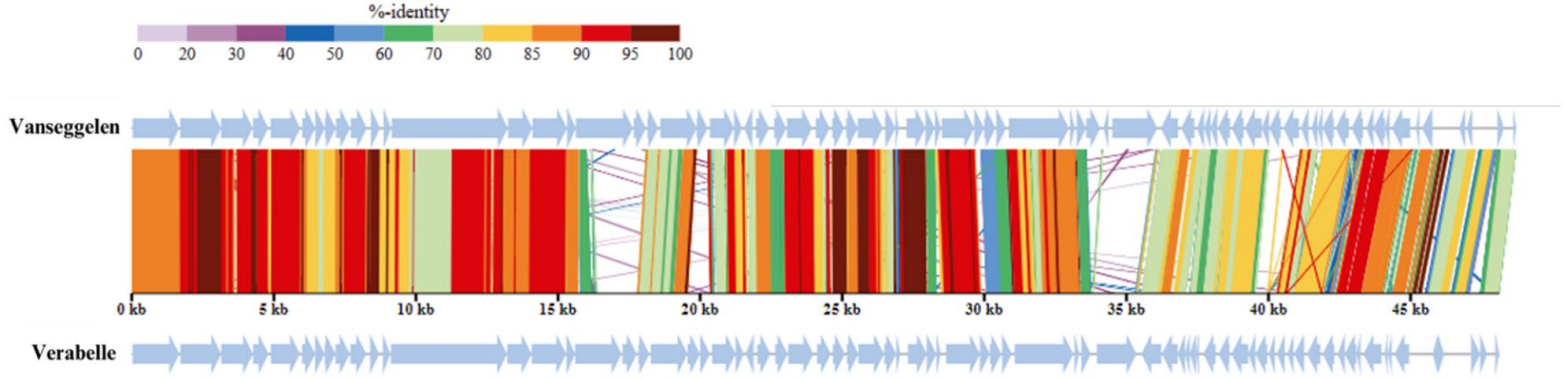
Genomic alignment of Vanseggelen and Verabelle. The predicted genes are indicated and the colored vertical blocks between the genomes indicate the level of nucleotide similarity. The genome alignment was generated through ViPTree^17^.

Whole genome sequencing and de novo assembly of *Streptomyces* phage Vanseggelen revealed a double-stranded DNA genome of 48,720 base pairs with a G + C content of 65.6% and a 3′-cohesive termini of CGGTACGTGAT. This genome contains 72 potential coding sequences (CDSs), of which 37 encode for hypothetical proteins and the function of 35 CDSs could be predicted (Fig. 6a). Verabelle revealed a slightly smaller double-stranded DNA genome of 48,126 base pairs with G + C content of 65.0% and a 3′-cohesive termini of CGTACCGTCAT. Again, the genome contains 72 potential CDSs, of which 40 are hypothetical proteins and the function of 32 CDSs could be predicted (Fig. 6b). The three additional CDSs that could be predicted in Vanseggelen are an additional minor tail protein, an RNA-polymerase sigma factor and a nucleoid associated Lsr2-like protein. Both genomes possess a putative integrase, indicating the capability of a temperate lifestyle. The Actinobacteriophage Database places both phages in Cluster BD, Subcluster BD3^9^. Although the genomes of both phages are similar, there are some sequence variations that result in differences at the protein level (Fig. 7).

**Figure 8:**
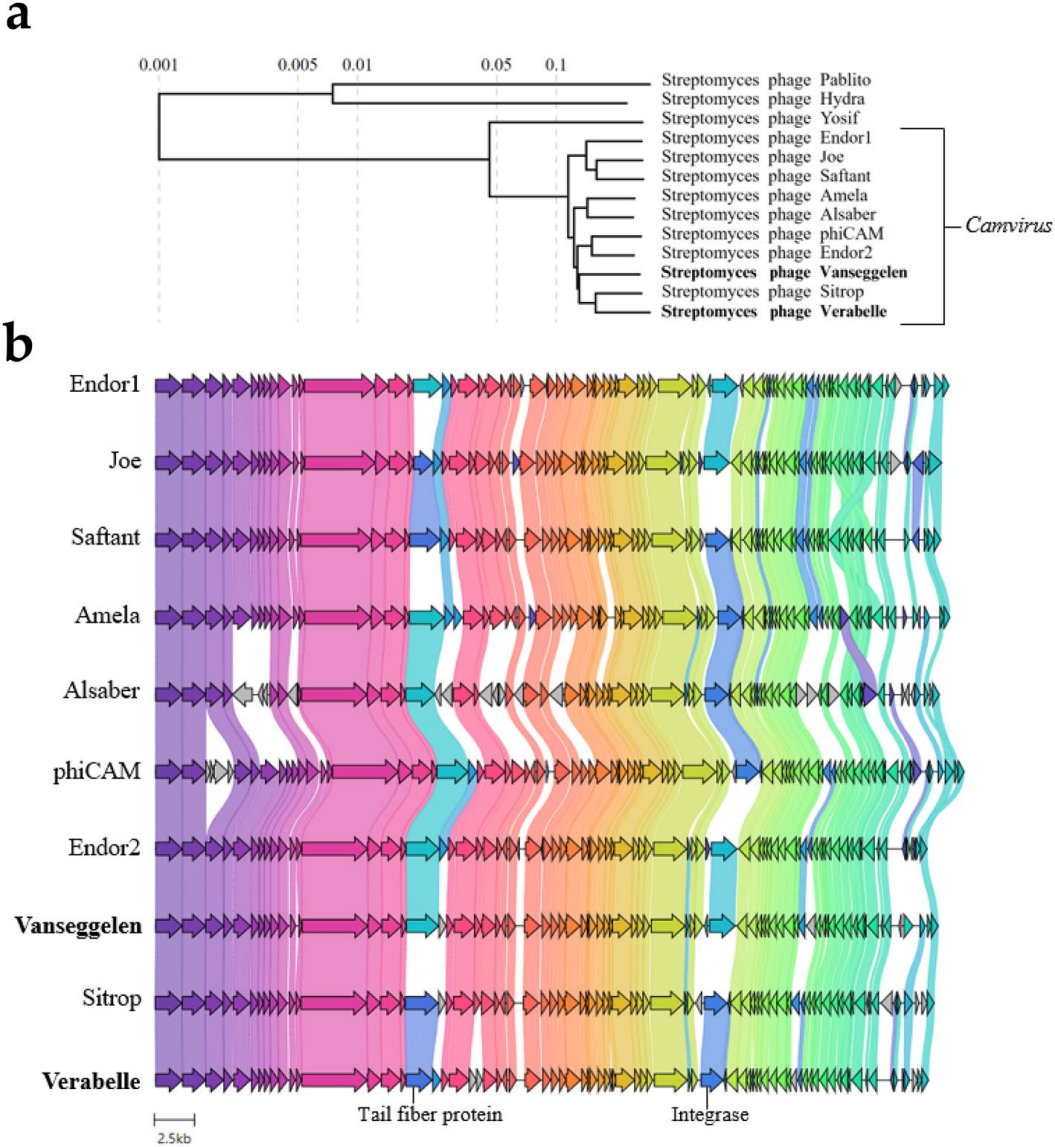
Phylogeny and genome similarity of Vanseggelen and Verabelle based on the whole genome sequences. (**a**) *Streptomyces* phages Vanseggelen and Verabelle belong to the genus *Camvirus*, within the subfamily *Arquattrovirinae*. The viral proteomic tree was generated through ViPTree^17^. (**b**) Genome alligment of all phages within the *Camvirus* genus starting at the terminase. Vanseggelen and Verabelle don’t share similarity in the tail fiber protein and integrase as indicated by light blue arrowheads in Vanseggelen and dark blue in Verabelle.

Phylogenetic analysis (Fig. 8a) based on the whole genome sequences of the closest-related phages, revealed that Vanseggelen is most closest related to *Streptomyces* phage Endor2 and Verabelle is most closely related to *Streptomyces* phage Sitrop. They are more distantly related to *Streptomyces* phages Pablito, Hydra and Yosif, which belong to different genera. These results indicate that both phages belong to the same genus, namely *Camvirus*. The genomic alignment of all phages within the *Camvirus* genus (Fig. 8b) show that phage Vanseggelen shares a similar tail fiber protein with phage Endor2, phiCAM, Alsaber, Amela and Endor2, while Verabelle shares a similar tail fiber protein with phage Sitrop, Saftant and Joe. The lack of genome similarity in tail fiber proteins between phage Vanseggelen and Verabelle might explain their difference in host range as seen in Tables 1 and 2. Additionally, phage Vanseggelen and Verabelle have a different integrase protein, indicating that they will likely integrate at different places in their host.

**Figure 9:**
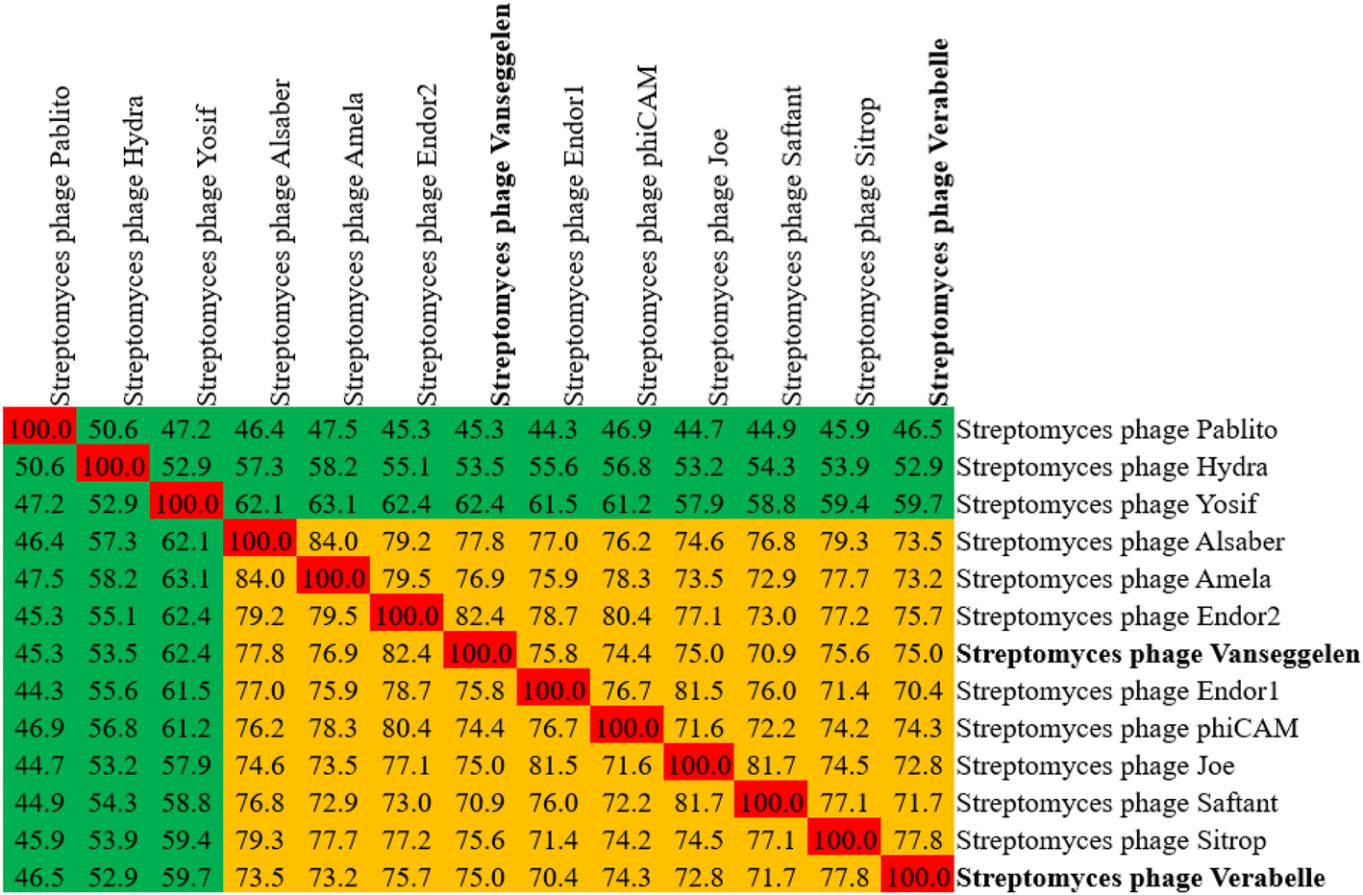
Intergenomic relatedness. Genomes of the top ten most similar *Streptomyces* phages Alsaber, Amela, Endor2, Endor1, phiCAM, Joe, Saftant and Sitrop were used to show relatedness. The genomes of *Streptomyces* phages Pablito, Hydra and Yosif are classified in different genera as shown in green. The heatmap was calculated by VIRIDIC^19^.

Next, a heatmap of the intergenomic relatedness (Fig. 9) was made. The two phages show a relatedness of 75.0 to each other and Vanseggelen is most related to phage Endor2 with a relatedness of 82.4, while Verabelle is most related to phage Sitrop with 77.8. The Bacterial Viruses Subcommittee of the International Committee on Taxonomy of Viruses has determined that if a phage exhibits ≥ 70% and < 95% nucleotide relatedness, the phage belongs to a new species in an undefined or existing genus. A sequence similarity of ≥ 95% is the cut-off for identifying a phage as a new species. Since the relatedness of Vanseggelen and Verabelle is lower than 95% compared to the top ten most similar *Streptomyces* phages, these phages are both considered as new species according to the International Committee on Taxonomy of Viruses (ICTV)^21^. A taxonomic proposal has been submitted to ICTV to officially recognize Vanseggelen and Verabelle as part of the genus *Camvirus,* as a new species called “*Camvirus vanseggelen*” and “*Camvirus verabelle*”.

## Discussion

In this study, the newly isolated *Streptomyces* phages Vanseggelen and Verabelle have been fully characterized and sequenced. These results increase our insights on phage-host dynamics in *Streptomyces* and expand available, sequenced and characterized *Streptomyces* phages. TEM images showed that both phages have a long, flexible non-contractile tail with an icosahedral capsid head typical for siphoviruses. Both phages belong to the class *Caudoviricetes,* which was confirmed by whole genome sequencing. Since the majority of the sequenced *Streptomyces* phages have a long non-contractile tail, this tail morphotype is possibly necessary for effectively penetrating the thick peptidoglycan layer of Gram-positive bacteria. The host range analysis showed that both phages can form lysis zones with a turbid morphology with some host strains, indicating that Vanseggelen and Verabelle are likely able to switch from a lytic to a lysogenic lifecycle. Indeed, since all other phages belonging to the genus *Camvirus* are temperate phages and whole genome sequencing revealed that both genomes contain an integrase protein, we can conclude that Vanseggelen and Verabelle are temperate phages as well^22^.

Of all sequenced *Streptomyces* phages available in the Actinobacteriophage database^9^, we have calculated that the average genome length is 73,556 bp with a G + C content of ~ 61%, which is relatively high, and could be explained by the relatively high G + C-content of their bacterial hosts. Both Vanseggelen and Verabelle have a double-stranded DNA with a genome length of 48,720 bp and 48,126 bp, which is a bit smaller than average. Both phages were able to infect several different *Streptomyces* strains, but could not infect *K. virifidaciens. Kitasatospora* is the sister genus of *Streptomyces* that also belongs to the phylum *Actinobacteria.* Their morphology and lifecycle are similar to *Streptomyces* bacteria, but *Kitasatospora* is usually identified by the fact that they cannot be infected by *Streptomyces* phages^23,24^. Possibly, because of the difference in cell wall composition, since the cell wall of *Kitasatospora* strains contain both LL- and meso-diaminopimelic acid^25^. It would be interesting to isolate and characterize phages on *Kitasatospora* species in the future to dissect differences in infectivity between *Streptomyces* and *Kitasatospora* phages.

Despite the high degree of nucleotide similarity between the genomes of Vanseggelen and Verabelle, there were sequence variations that resulted in differences at the protein level, which could also account for the differences in host range. *Streptomyces* phage Verabelle showed a slightly broader host range infectivity than Vanseggelen, which could be explained by potential phage specific defense mechanisms or the difference in tail fiber proteins. Additionally, a different integrase protein was found, which suggest that both the tail fiber protein and integrase are a common source of variation in closely related phages within the *Camvirus* genus.

The one-step growth curve of Vanseggelen and Verabelle showed a long latent period of 140 min and a relatively low burst size, even when compared to other Streptomyces phages^12,20^. The low burst size could be the result of the morphological complexity of *Streptomyces*^20^. The meaning of MOI loses its significance once spores have germinated and a mycelium is formed as multiple phages are able to attach, but the mycelium would still be counted as one colony forming unit^15,26^. This notion of MOI in *Streptomyces*-phage dynamics complicates quantitative experiments and could explain the low burst sizes shown in this study.

Temperature plays a fundamental role in phage attachment, penetration, multiplication, and the length of the latent period, and is therefore a crucial factor for phage viability^27^. Both phages remain stable at temperatures ranging from 30 to 45 °C while phage viability decreases at higher temperatures. These results are consistent with a previous study that shows that phages most likely lose their DNA from the capsid head between temperatures of 50 and 60 °C due to the tail complex breaking of the capsid head^28^. This results in the marked loss of infectivity and structural changes in the capsid proteins. It is suspected that denaturation of DNA and proteins within the capsid head only occurs at temperatures above 80 °C^29^. Another important factor for phage viability is the acidity of the environment in which the phage persists. *Streptomyces* strains can grow well between a pH range of 5.5–11.5^28^. However, both Vanseggelen and Verabelle remain stable at pH values from 4.0 to 10.0. Although the exact effect of acidity on the capsid head or tail has not been clearly elucidated yet, a previous study showed that extreme pH values, such as highly acidic or highly alkaline conditions, can result in the denaturation or structural changes to phage capsid head proteins^30^.

The *Streptomyces* phages described in this study can help to discover new insights in the interactions between *Streptomyces* bacteria and bacteriophages. More fundamental research is needed to study the attachment, lifecycle and infection of phages targeting multicellular bacteria, like *Streptomyces.* Since *Streptomyces* are producing a wide range of clinically relevant antibiotics, such studies become increasingly important in order to keep exploiting *Streptomyces*’ antimicrobial potential and abolish phage infection in various industries like biotechnology, agriculture and medicine.

## Data Availability

The genomes of *Streptomyces* phages Vanseggelen and Verabelle are available on NCBI (GenBank Accession No. OQ970438 and OQ970439). Both phages are available at the Dutch “Fagenbank” in Delft.
